# Calcified Amorphous Tumor and Granulomatosis with Polyangiitis—Case Report and Systematic Review of the Literature

**DOI:** 10.3390/jcm14010084

**Published:** 2024-12-27

**Authors:** Mathieu N. Suleiman, Abbas Agaimy, Oliver Dewald, Ann-Sophie Kaemmerer-Suleiman, Fritz Mellert, Michael Weyand, Frank Harig

**Affiliations:** 1 Department of Cardiac Surgery, University Hospital Erlangen, Friedrich-Alexander-University Erlangen-Nürnberg, 91054 Erlangen, Germany; oliver.dewald@uk-erlangen.de (O.D.); ann-sophie.kaemmerer@uk-erlangen.de (A.-S.K.-S.); friedrich.mellert@uk-erlangen.de (F.M.); frank.harig@uk-erlangen.de (F.H.); 2 Institute of Pathology, University Hospital Erlangen, Friedrich-Alexander-University Erlangen-Nürnberg, 91054 Erlangen, Germany; abbas.agaimy@uk-erlangen.de

**Keywords:** calcified amorphous tumors, CAT, cardiac mass, granulomatosis with polyangiitis (GPA), Wegener’s granulomatosis

## Abstract

**Background:** Calcified amorphous tumor (CAT) is a rare, mostly incidental tumor-like cardiac lesion of unknown histogenesis. Current imaging modalities do not differentiate between CAT and other masses. As it can be a source for embolization, surgical excision of CAT is mandatory. CAT in patients with Granulomatosis with polyangiitis (GPA) is exceedingly rare. **Methods:** This systematic literature review was prompted by the case of a CAT in a patient with GPA. The search of all types of studies in two databases (PubMed and Scopus) was conducted through November 2024 to identify the relevant studies. **Results:** Nine studies were included describing cases of patients being diagnosed with GPA and a cardiac mass. All included patients had a histopathological examination of the either biopsied or surgically resected mass. Only one case reported a CAT. In our case, the patient was diagnosed with GPA through a kidney biopsy, whereas a cardiac mass in the right atrium was diagnosed by echocardiography during evaluation for possible kidney transplantation. One year later a progression was observed, and the mass was resected. The histopathological examination revealed a CAT. The patient could be successfully discharged in a good clinical condition. **Conclusions:** This systematic literature search and case report highlight the importance of regular echocardiographic examination in patients with GPA. Moreover, surgical excision is crucial for the diagnosis and for further therapy planning, regardless of whether the mass is neoplastic or not.

## 1. Introduction

Calcified amorphous tumors of the heart (CATs) are rare and intriguing entities within cardiology. These non-neoplastic intracardiac tumors were first described in 1997 by Reynolds et al., marking the beginning of their recognition in the medical community [1]. Histologically, CATs are characterized by the presence of acellular fibrinous amorphous material and calcifications, distinguishing them from other types of cardiac tumors. This unique histological composition can complicate diagnosis and treatment, requiring a careful and nuanced approach [2].

CATs are most frequently localized on the mitral valve or the mitral valve annulus, accounting for 36% of cases. They can also be found in the right atrium (21%), right ventricle (17%), left ventricle (14%), left atrium (7%), and on the tricuspid valve or tricuspid valve annulus (5%) [2]. This distribution pattern suggests a tendency for CATs to affect various parts of the heart, potentially leading to a range of clinical presentations, depending on their location. CAT is found incidentally, in association with cerebral infarction and acute heart failure, among others [3].

Granulomatosis with polyangiitis (GPA), also known as Wegener’s disease, adds another layer of complexity when considering cardiac conditions. First described in 1936 [4], GPA is a rare systemic disease characterized by pauci-immune vasculitis and necrotizing granulomas, affecting small- and medium-sized vessels [5]. The combination of these two rare conditions—CAT and GPA—is exceedingly uncommon, and has scarcely been documented in the medical literature.

To the best of our knowledge, the occurrence of CAT in a patient with GPA is extremely rare. This rarity makes each case report valuable, as it can provide insights into the possible interactions between these conditions, their clinical manifestations, and potential treatment approaches. Therefore, this case report aims to contribute to a better understanding of the clinical course of this rare combination, potentially guiding future diagnosis and management strategies for similar cases. By documenting and analyzing this case, we hope to shed light on the complexities and challenges associated with managing patients who present with both CAT and GPA, ultimately improving patient care and outcomes.

## 2. Case Presentation

**Figure 1:**
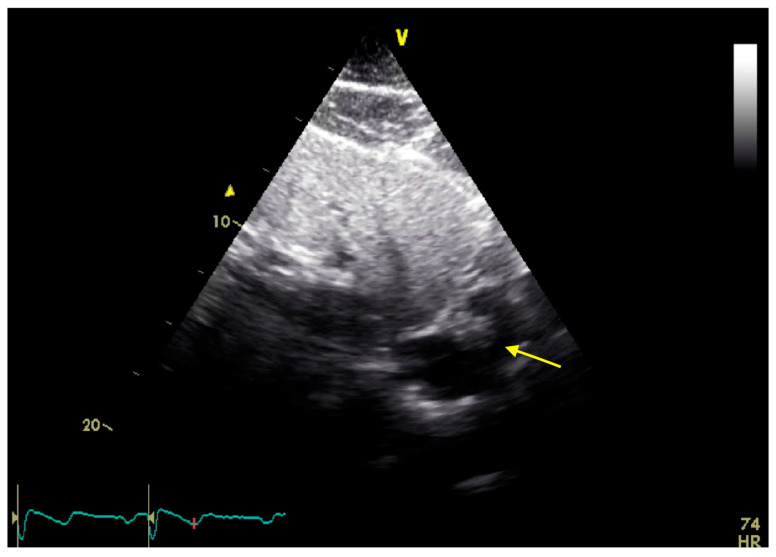
Transthoracic echocardiography in a subcostal view showing the CAT (yellow arrow).

**Figure 2:**
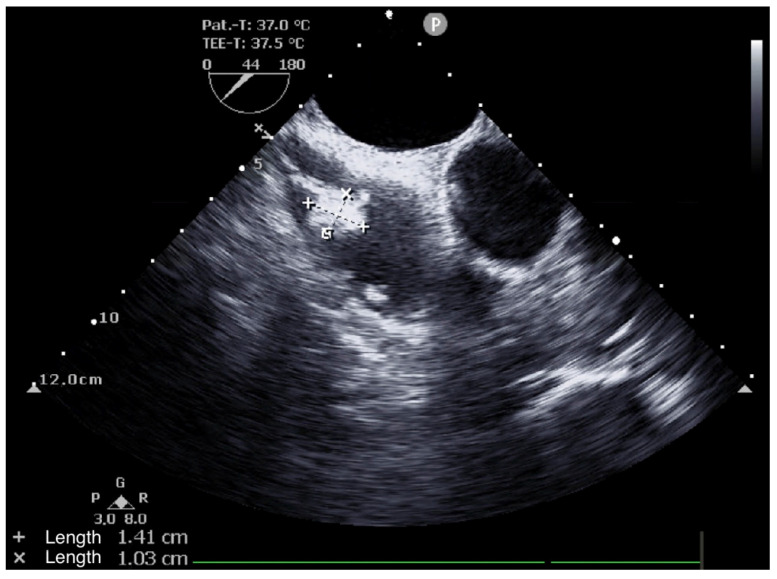
Pre-operative transesophageal echocardiogram (TEE), demonstrating the size of the right atrial (RA) mass.

**Figure 3:**
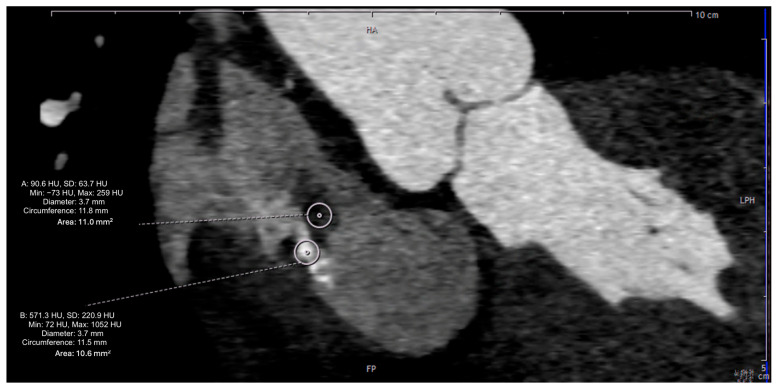
Cardiac CT image demonstrating tissue characterization using Hounsfield Units (HUs).

**Figure 4:**
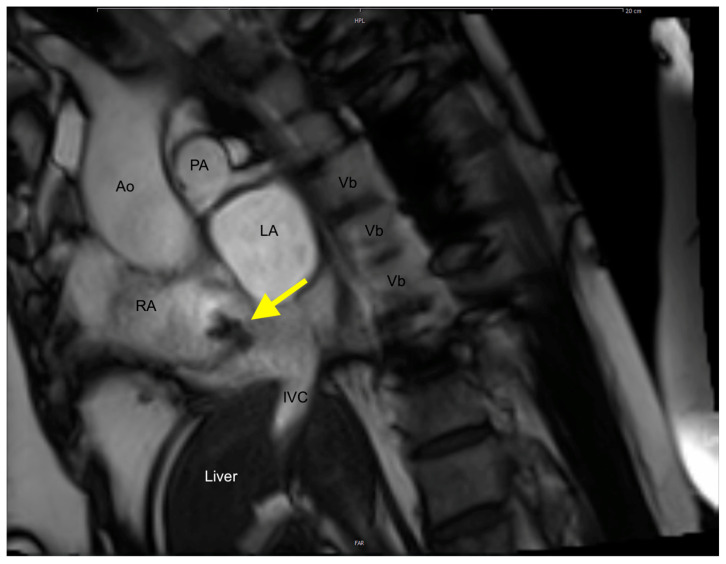
Cardiac MRI demonstrating the CAT in the right atrium (yellow arrow). (Ao, Aorta; IVC, Inferior Vena Cava; LA, Left atrium; PA, Pulmonary artery; RA, Right Atrium; Vb, Vertebra).

A 65-year-old woman (162 cm, 77.5 kg, BMI: 29.5 kg/m^2^) presented to an emergency department of a regional hospital with dyspnea, hemoptysis, vomiting and fatigue for four weeks. The patient’s past medical history included arterial hypertension, impaired kidney function and renal anemia with secondary hyperparathyroidism. Shortly after admission, the patient developed acute renal failure with anuria. A kidney biopsy showed a PR3-ANCA positivity, and GPA was diagnosed. After initiation of an immunosuppressive therapy with Cyclophosphamide (CyC) and corticosteroids, followed by a maintenance treatment with Rituximab (RTX) and prednisolone, the hemodialysis was initiated. One year later, the patient presented for evaluation for kidney transplantation, including a transthoracic echocardiography (TTE), transesophageal echocardiography (TEE), and a computed tomography, as well as an MRI (Figure 1, Figure 2, Figure 3 and Figure 4). TEE revealed an 8 × 5 mm sclerotic structure in the right atrium.

A few months later, the patient presented again in the regional hospital with rib pain and dyspnea. Clinical examination did not reveal any signs of heart failure or focal neurological deficits. During the physical examination, the patient was normotensive, slightly arrhythmic due to single supraventricular extra beats, with normal heart sounds and without pathologic heart murmurs.

As pulmonary embolism was suspected, heparin was administered intravenously and pulmonary perfusion scintigraphy was performed, depicting signs of pulmonary embolism on the right sided postero- and antero-basal lower-lobe segment.

Transesophageal echocardiography (TEE) confirmed the foreknown, non-floating, pedunculated mass with hyperechogenic, hypoechogenic components and presumably calcification in the right atrium. However, the size of the right atrial structure had meanwhile increased to 10 × 18 mm. For further assessment of the RA structure, a cardiac computed tomography scan (CT) was performed. Since the CT did not provide any additional relevant diagnostic information, an additional contrast-enhanced Magnetic Resonance Scan (MRI) was performed. MRI visualized a partially soft-tissue dense, partially calcified tumor, adherent to the dorsolateral wall of the right atrium, without contrast-media uptake in perfusion and with contrast-media uptake in late imaging. The most likely diagnosis from imaging was a partially calcified atrial myxoma, partly embolizing into the pulmonary vascular bed.

A preoperative TEE showed the RA structure to be attached to the Eustachian valve at the junction of the RA and the IVC (Figure 2).

**Figure 5:**
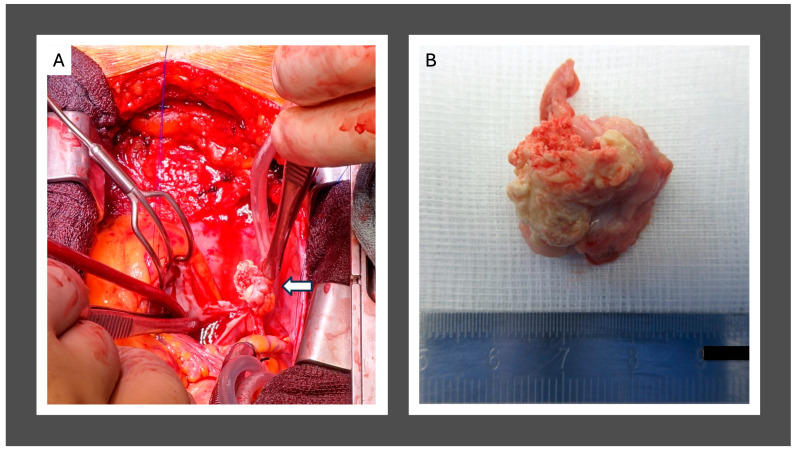
Intra-operative exposition of the CAT. Arrow indicates the tumor (**A**); in toto resection of the CAT, ex situs (**B**).

**Figure 6:**
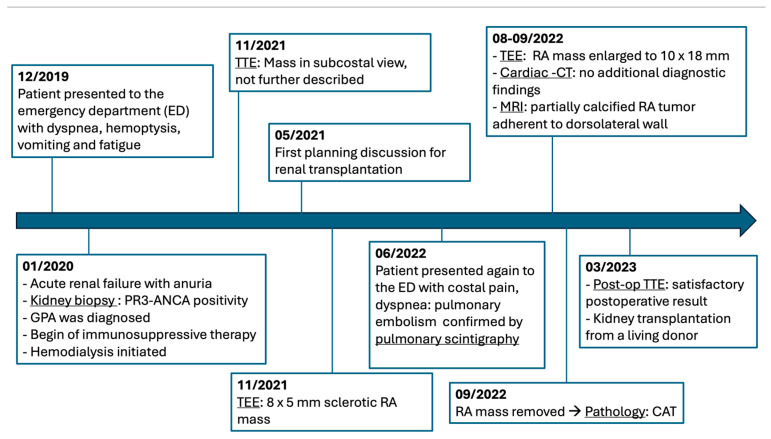
Detailed timeline of the patient’s diagnostic and therapeutic journey (CAT, calcified amorphous tumor; CT, computed tomography; GPA, glomerulonephritis with polyangiitis; MRI, Magnetic Resonance Imaging; PR3-ANCA, proteinase 3 antineutrophil cytoplasmic antibodies; RA, right atrium; TEE. transesophageal echocardiography; TTE, transthoracic echocardiography).

Based on the available findings, cardiac surgery was deemed indicated. After median sternotomy, the right atrium was opened, and a whitish tumor of approximately 20 × 10 mm with a rough surface was seen. The tumor was removed in toto and macroscopically in sano (Figure 5). Subsequently, the RA atriotomy was closed with a xenogenic pericardial patch. After thoracic closure, the patient was transferred to the ICU with an uneventful postoperative course. Because of the previous pulmonary embolism, the patient was discharged with initiated Warfarin therapy (Figure 6).

**Figure 7:**
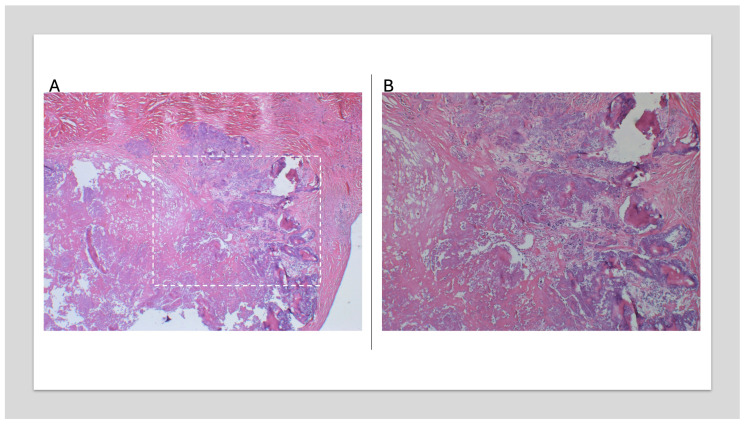
(**A**) Tumefactive dystrophic calcifications embedded within fibrous tissue rich in degenerated fibrinous material (H&E stain; ×100). Dotted area depicts higher magnitude (**B**). (**B**) Details of the granular calcified tissue (H&E stain; ×200).

**Figure 8:**
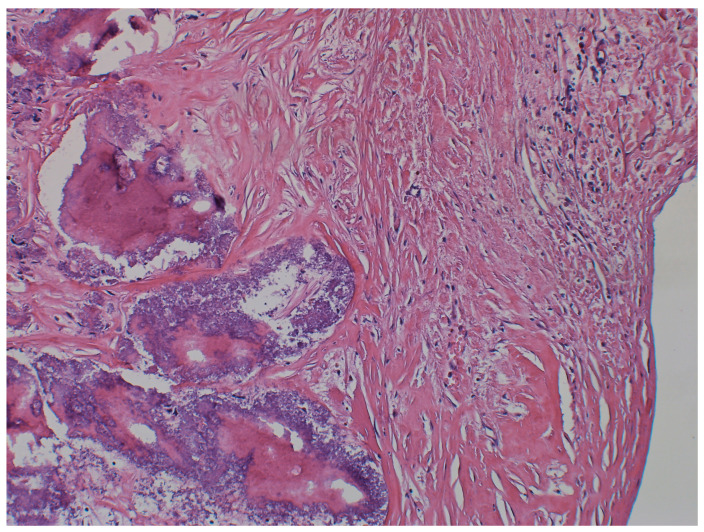
Higher magnification of the findings (H&E stain; ×400).

Microscopic examination (Figure 7 and Figure 8) of the excised tumor showed tumefactive dystrophic calcifications embedded within fibrous tissue and degenerated fibrin. Adjacent myocardial tissue revealed minor caliber variations of the cardiomyocytes without cellular atypia or evidence of inflammation or vasculitis. Findings were consistent with a benign pseudoneoplastic lesion (CAT).

A follow-up transthoracic echocardiogram (TTE) was performed after 5 months. The left ventricle (LV) showed normal dimensions with an estimated ejection fraction (EF) of 55%, indicating normal systolic function, without clear regional-wall motion abnormalities. The right ventricle (RV) was of normal size and contractility. Both atria were of normal size. The aortic valve (AV) and mitral valve (MV) showed no significant valvular abnormalities, while the tricuspid valve (TV) exhibited mild insufficiency. Systolic pulmonary arterial pressure (sPAP) was 18 mmHg, with additional central venous pressure (CVP). The pulmonary arteries (PAs) appeared unremarkable within the visible range. No pericardial effusion was observed, and the inferior vena cava (IVC) was of normal width with respiratory modulation. No pleural effusions were detected. Overall, the postoperative results were satisfactory.

Six months after the cardiac operation, the patient successfully received a kidney transplant from a living donor.

## 3. Methods

### 3.1. Systematic Review—Search Strategy

The search strategy was conducted in accordance with the PRISMA-S extension of the PRISMA statement for reporting literature searches in systematic reviews. We followed the Preferred Reporting Items for Systematic Reviews following Meta-Analyses (PRISMA) guidelines.

The following databases were systematically searched: Pubmed and SCOPUS, using the search terms “GPA” OR “granulomatosis with polyangiitis” OR “Wegener`s granulomatosis” AND “cardiac mass” OR “calcified amorphous tumor” (Table 1).

We included all articles published in English prior to November 2024 (Supplementary Figure S1). All included articles were identified by two authors (MS and ASK). Afterwards, the full texts of all selected studies were reviewed. No study registries or other online resources were searched. No date, language or study-design filters were used. No additional studies were sought by contacting authors.

### 3.2. Study Quality Assessment

The quality of the included studies was evaluated by MS, ASK and FH.

Consent for publication was obtained from the patient.

### 3.3. Data Extraction

Studies were considered eligible for inclusion if they (1) involved individuals with the diagnosis of GPA and a cardiac mass, (2) were published in English and (3) had a pathological examination of the resected or biopsied cardiac mass. In total, 9 cases were included. Conversely, studies were excluded if they (1) were duplicate publications, (2) did not report patients having the diagnosis of GPA and a cardiac mass, or (3) had no histopathological examination of the resected or biopsied cardiac mass.

All original searches were conducted on 8 November 2024.

## 4. Results

In total, 29 unique citations were identified from SCOPUS and 99 in PubMed after an initial search. Of these 128, 55 were excluded after titles and abstracts were screened, mainly because they were, by title, unrelated to the current study. Then, the full text of 67 articles was reviewed, 61 of which were excluded. A total of four reports were included in the review by matching the criteria.

Additional articles were manually identified by screening the cited references. A total of 160 references were checked, and 49 reports were sought for retrieval. Afterwards, 44 were excluded by irrelevance or duplicity. Five reports matched the criteria of the search, and were included in the review. The data were published between 1980 and 2023, involving a total of nine patients.

Finally, a total of nine studies were eligible and included in the systematic review. The characteristics of the nine included studies are summarized in Table 1. Three studies (33.3%) were conducted in European countries, namely Germany, Sweden and Poland, whereas six (66.7%) studies were in non-European countries, namely the USA and Japan. All studies sampled patients with GPA from clinic-based populations. All patients reported suffered from GPA and had a histopathological examination of the cardiac mass. The studies’ characteristics are summarized in Table 1. The presented cases of Tasken et al. [6] and Ferns et. al. [7] 2010 had a biopsy of the cardiac mass; all other cardiac masses were surgically removed. Only the case of Fukushima et al. [3] had a histopathologically confirmed CAT.

**Table 1:** Clinical characteristics of published cases.

Report	Age (Years)/Sex	Diagnosis of GPA	Pathology	Location of Mass	Removal
1. Kosovsky et al., 1991 [8]	16, M	By left Caldwell–Luc procedure and maxillary sinus mucosal biopsies	Acute and organizing granulomatous vasculitis involving the myocardium	Upper 2/3 of the anterior right ventricular wall	Surgical resection
2. Ferns et al., 2010 [7]	14, F	Positive C-anti-neutrophil cytoplasmic antibody pattern, renal biopsy	Necrotic tissue with marked inflammatory infiltrate consisting primarily of neutrophils and eosinophils, additional necrosis and fibrosis of adjacent myocardial tissue	Left ventricular apex and adherent to the left ventricular trabeculations	Biopsy
3. Attaran et al., 2010 [9]	52, M	30-year history of GPA	Features of acute and chronic inflammation, myxoid degeneration, prominent eosinophilic infiltration, and some small blood vessels showing intimal fibrosis	Large circular mass involving the anterior mitral leaflet on the atrial side and involvement of the papillary muscles and aortic valve	Surgical resection
4. Herbst et al., 2003 [10]	56, F	Due to clinical findings and pathology of the cardiac mass	Intense inflammatory infiltrate with aggregates of neutrophils and eosinophils infiltrating the collagen and forming small micro-abscesses surrounded by histiocytes, lymphocytes, and plasma cells; inflammatory process infiltrated adjacent myocardium and produced a reactive myocyte atypia	Mass involving most of the anterior leaflet of the mitral valve, as well as fibrous aortic continuity	Surgical resection
5. Fukushima et al., 2022 [3]	80, F	History of GPA	Nodules of calcification and fibroblasts; CAT	Anterior mitral leaflet	Surgical resection
6. Oliveira et al., 2005 [11]	63, F	History of nasal GPA	Granulomatous tissue	Left ventricular outflow tract	Surgical resection
7. Corin et al., 2022 [12]	58, F	Positive Proteinase 3 antineutrophil cytoplasmic antibodies (PR3-ANCA)	No atypia, scattered cells in a faint myxoid stroma and ring-shaped structures around vessels, confirming atrial myxoma	Right atrial mass	Surgical resection
8. Harris et al., 2012 [13]	14, F	Renal biopsy	Necrotic tissue with acute inflammation consisting of neutrophils and eosinophils with absence of granulomas	Apex of the left ventricle	Surgical removal
9. Taskesen et al., 2015 [6]	53, F	Autoimmune serology: positive for anti-myeloperoxidase antibodies; negative antinuclear antibodies, classic antineutrophilic cytoplasmic antibodies, and anti-proteinase 3 antibodies	Mixed lymphoplasmacytic and neutrophilic inflammation, poorly formed granulomas	Infiltration of interatrial and interventricular septae, both atria, and left ventricular outflow tract and ventricular side of the mitral valve anterior leaflet	Biopsy

F, female; M, male; GPA, glomerulonephritis with polyangiitis; CAT, calcified amorphous tumor; ANCA, antineutrophil cytoplasmic antibodies.

## 5. Discussion

To our knowledge, this is an extremely rare case of calcified amorphous tumor (CAT) of the right atrium in a patient with granulomatosis with polyangiitis (GPA).

CAT was first described by Reynolds et al. in 1997 [1]. CAT is most commonly an incidental finding, and current imaging modalities do not differentiate between CAT and other masses [14].

It can be a rare source of pulmonary embolization, as in the presented case, or systemic embolization, which was described in more than 30% of reported cases [2,15].

The etiology of CAT is poorly understood. However, CAT has already been described in the context of end-stage renal disease/hemodialysis [16,17,18].

GPA is a rare chronic disorder that affects multiple organs, including the kidneys, lungs, and both the upper and lower respiratory tracts [19]. Cardiac involvement occurs in 6–25% of unselected patients with GPA, showing a variety of abnormalities, including intracardiac masses [4,12,20]. However, heart involvement is uncommon in GPA, with conditions like pericarditis, myocarditis, or conduction system issues being more typical [21]. Studies suggest that cardiac masses in GPA are usually inflammatory, rather than tumorous [12]. Additionally, there are reports of GPA cases occurring alongside cardiac myxoma [12].

Histologically, a cardiac amorphous tumor is characterized by nodular calcifications in the presence of fibrin. Degeneration and focal chronic inflammation may be present, as well [1,2].

Several factors may be linked with CAT, such as endothelial damage, blood stasis, hypercoagulability states, abnormal calcium metabolism and chronic inflammation. End-stage kidney disease and hemodialysis may lead to the development of these factors. Indeed, chronic kidney disease is associated with metabolic bone disease and ectopic calcification via disruption of calcium homeostasis and alterations of physiological calcium regulatory mechanisms, such as abnormal regulation of parathyroid hormone and vitamin D [16]. This may also be associated with mitral annulus calcification, which was not the case in our patient [17].

Different pathologies can be found in the right atrium. Differential diagnosis of masses in the RA includes benign and malignant tumors, such as myxomas or sarcomas, entities simulating cardiac tumor, such as thrombosis/vegetations involving the tricuspid valve, the valvula eustachii, rete chiari, and valvula thebesii (valvula sinus coronarii). In particular, the latter are often misinterpreted as tumors [22].

The evaluation of cardiac masses requires precise imaging for accurate diagnosis and treatment planning. Magnetic Resonance Imaging (MRI) is a key modality, due to its superior soft-tissue differentiation, ability to assess vascularity and perfusion with gadolinium contrast, and capacity for functional imaging [23,24]. These features make it particularly effective in distinguishing benign from malignant lesions and determining the impact of masses on cardiac function. MRI’s high spatial resolution also aids in surgical planning, and its radiation-free nature is advantageous for repeated imaging [25].

However, MRI is not without limitations. It is less accessible, more costly, and contraindicated in patients with certain devices or severe claustrophobia. Additionally, it requires longer imaging times and is less effective at detecting calcifications compared to computed tomography (CT). Other modalities, such as transthoracic and transesophageal echocardiography, provide complementary information, with transthoracic echo (TTE) being widely available and transesophageal echo (TEE) offering high-resolution imaging, also of posteriorly located masses.

While MRI is often the cornerstone for cardiac mass evaluation, a multimodal approach incorporating echocardiography and CT ensures accurate diagnosis and optimal outcomes by leveraging the strengths of each imaging technique [26].

From these masses, symptoms can arise, including blood flow obstruction, depending on where the tumor is located or has embolized, as in the given case, where pulmonary embolism occurred. Regardless of whether the mass is neoplastic or not, surgical excision is crucial for the diagnosis and for further therapy planning [1].

The use of anticoagulation or antiplatelet therapy after cardiac tumor removal is critical for preventing thromboembolic complications, particularly in patients with prior embolic events or conditions that increase thrombosis risk [27].

After cardiac surgery, however, the decision to initiate anticoagulation must carefully balance the thrombotic risk against the potential for bleeding, which is heightened in the early postoperative period. Better controllable agents, like low-molecular-weight heparin, may be used temporarily to manage this transition.

In the long term, for benign tumors, such as calcified amorphous tumors or atrial myxomas, anticoagulation may not always be necessary, unless there is a documented history of embolism or atrial fibrillation. When prosthetic materials are used in surgery, anticoagulation is often recommended, to prevent thrombus formation.

The duration of anticoagulation or antiplatelet therapy depends on the patient’s individual risk factors and surgical findings. While some patients may only require short-term therapy, others with persistent risk factors, such as atrial fibrillation or prosthetic materials, may need long-term or lifelong anticoagulation.

In any case, in patients with cardiac tumors, careful multidisciplinary collaboration of cancer-associated thrombosis is essential to tailor therapy and ensure optimal outcomes, while minimizing the risks of bleeding and thromboembolism [28].

Therefore, although patients with calcified amorphous tumors (CATs) who undergo surgical excision often have a favorable prognosis, it is essential to maintain vigilant follow-up and to deepen our understanding of the intricate relationships between this rare pathology and other comorbid conditions. This highlights the critical need for interdisciplinary collaboration, as insights from various specialties can help refine diagnostic approaches, optimize treatment strategies, and ultimately improve patient outcomes. Continued research efforts, especially in cardiovascular medicine, are vital for advancing our knowledge of CAT and similar uncommon conditions, providing a foundation for improved patient care and more comprehensive guidelines in the future.

## 6. Limitations

A major limitation of this study is the limited literature available on this rare condition, which makes it challenging to perform thorough comparisons and fully understand its clinical presentation, management strategies, and outcomes. The small number of reported cases restricts the ability to generalize findings, and the absence of standardized diagnostic and therapeutic guidelines may result in inconsistencies in patient care. Additionally, the inherently small sample size associated with rare diseases reduces the statistical strength of our observations. More research, ideally through multicenter collaborations and extended follow-up studies, is needed to gain a clearer understanding of this condition. Moreover, the complex relationship between CAT and systemic diseases such as granulomatosis with polyangiitis (GPA) adds another layer of complexity, highlighting the need for further investigation into the mechanisms and clinical implications of this uncommon association.

## 7. Future Directions

Future research could focus on molecular and genetic profiling, including the identification of biomarkers. Establishing registries or conducting multicenter studies would also help track patient outcomes, identify factors associated with recurrence and survival, and enhance our understanding of these conditions. Furthermore, investigating new therapeutic options, such as targeted molecular therapies, immunotherapy, and advanced surgical techniques, should be considered as potential avenues for improved treatment strategies.

## 8. Conclusions

Cardiac calcified amorphous tumors (CATs) are rare, benign pseudotumors that are frequently misdiagnosed due to their unusual presentation and resemblance to other cardiac masses. Despite their non-malignant nature, CATs have the potential to cause embolic events, making surgical excision essential for both treatment and prevention of complications. Generally, the prognosis following surgery is positive, as the removal of the tumor is often curative. However, this condition remains poorly understood, especially when it occurs alongside systemic diseases, such as granulomatosis with polyangiitis (GPA), where it presents unique diagnostic and management challenges. In patients with GPA, regular cardiac monitoring through echocardiography is advisable, to detect the presence of any cardiac masses or track the progression of pre-existing cardiac abnormalities. Such surveillance is crucial, as the coexistence of CAT and GPA introduces additional complexities, potentially impacting both prognosis and treatment approach. Following the resection of a cardiac CAT, lifelong follow-up with a cardiology specialist is recommended, to monitor for recurrence or other related complications, ensuring sustained patient well-being and enabling timely intervention if new cardiac issues arise. Further research into the mechanisms linking CAT and systemic diseases like GPA is needed to improve patient care and develop clearer guidelines for managing these rare and complex cases.

## Data Availability

Not applicable.

## Supplementary Materials

The following supporting information can be downloaded at: https://www.mdpi.com/article/10.3390/jcm14010084/s1, Figure S1: PRISMA Flow Diagram. Reference [29] is cited in the supplementary materials.
